# News and Events of the Week

**Published:** 1911-09-16

**Authors:** 


### NEWS AND EVENTS OF THE WEEK.

The Tuberculosis Congress which was to have been held in Rome on September 21 next has been postponed to April 1912.

The Council of the Borough of Stepney has issued an order placing measles among the notifiable diseases from September 9 till March 1912.

The Debrousse Hospital, founded at Sainte-Foy, Lyons, by Madame Debrousse at a cost of eight million francs (£320,000), has just been opened. It is entirely devoted to the care of the aged.

After being duly approved by the Local Government Board, and advertised, an order has been issued to the medical profession in the administrative County of London, by which the London County Council resolves that section 55 of the Public Health (London) Act, 1891, with respect to the notification of infectious disease shall apply, up to and including the 12th of March, 1912, to the disease known as acute poliomyelitis or acute encephalitis. This order applies only to cases of the disease beginning on or after September 1, 1911.

### SERUM-THERAPY IN THE FRENCH LAW COURTS.

A decision which cannot fail to excite comment has recently been given by the Criminal Chamber of the French Cour de Cassation. According to the law of 1895 pharmacists are forbidden to sell serums, but the restriction was not held to be applicable to physicians, who hitherto were able to make use of any serum their fancy dictated. As the result of the recent decree the law is now held to apply equally to physicians. A Parisian doctor who had been prosecuted for injecting a serum was acquitted both by the original court at which the case was tried and by the Court of Appeal. The public prosecutor then took the case to the Cour de Cassation and finally won his case. The magistrates of the supreme court have reversed the decision of the lower tribunals, thereby restraining the liberty of action of the profession in the matter of the use of serums. The physician in question will have to be put on his trial again, this time at another court.

